# Gut microbiota changes in horses with *Chlamydia*

**DOI:** 10.1186/s12866-023-02986-8

**Published:** 2023-09-02

**Authors:** Youshun Jin, Wei Li, Xuli Ba, Yunhui Li, Yanyan Wang, Huaiyu Zhang, Zhaocai Li, Jizhang Zhou

**Affiliations:** 1 State Key Laboratory for Animal Disease Control and Prevention, Key Laboratory of Veterinary Public Health of Agriculture Ministry Lanzhou Veterinary Research Institute, Chinese Academy of Agricultural Sciences, Lanzhou, 730046 China; 2 State Key Laboratory for Animal Disease Control and Prevention, College of Veterinary Medicine, Lanzhou Veterinary Research Institute, Lanzhou University, Chinese Academy of Agricultural Sciences, Lanzhou, 730000 China; 3 Animal Pathology Laboratory, College of Veterinary Medicine, Northwest A&F University, Xianyang, 712100 China

**Keywords:** Zoonoses, *Chlamydia*, Horse, Gut microbiota

## Abstract

**Background:**

Zoonotic diseases pose a significant threat to public health. *Chlamydia*, as an intracellular pathogen, can colonize the intestinal tract of humans and animals, changing the gut microbiota. However, only a few studies have evaluated alterations in the gut microbiota of horses infected with *Chlamydia*. Therefore, this study aimed to investigate gut microbiota and serum biochemical indicators in horses with *Chlamydial* infection (IG) and healthy horses (HG). Fecal and blood samples were collected from 16 horses (IG: 10; HG: 6) before morning feeding for the determination of gut microbiota and serum biochemical parameters.

**Results:**

The results showed that total globulin (GLB), alanine aminotransferase (ALT), and creatine kinase (CK) levels were significantly increased in IG compared with HG. Notably, the gut microbial diversity increased in IG compared with HG. Furthermore, *Moraxellaceae* and *Akkermanisa* abundance decreased in IG, while *Streptococcus*, *Treponema*, *Prevotella*, and *Paraprevotella* abundances (13 genera of bacterial species) increased. Compared with HG, carbohydrate metabolism increased in IG while amino acid metabolism decreased. In addition, the abundance of 18 genera of bacteria was associated with the level of five serum biochemical indicators.

**Conclusions:**

In summary, this study elucidated the influence of *Chlamydia* infection in horses on the gut microbiota, unraveling consequential alterations in its composition and metabolic profile. Therefore, this study improves the understanding of *Chlamydia*-induced intestinal infections.

**Supplementary Information:**

The online version contains supplementary material available at 10.1186/s12866-023-02986-8.

## Introduction

Zoonoses have attracted much attention globally due to their infectious nature. About 60% of known infectious diseases and 75% of emerging infectious diseases may be directly derived from zoonotic diseases [1, 2], indicating that zoonoses are a threat to global public health. A graver concern pertains to the annual global incidence of zoonotic infections, afflicting an estimated 2.5 billion individuals and resulting in an unfortunate mortality rate of nearly 2.7 million [3]. Emphasizing the imperative, the maintenance of optimal health status among intermediate host animals emerges as the quintessential determinant in preempting the cross-species transmission of zoonoses [4].

*Chlamydia* is an ancient zoonotic disease with more than 15 different species [5]. *Chlamydia* is obligate intracellular parasitic, Gram-negative bacteria with a unique biphasic developmental cycle [6, 7]. Although *Chlamydia* infects different hosts, the disease pathology is similar [8]. Persistent *Chlamydia* infection can lead to serious sequelae, such as miscarriage, pneumonia, and enteritis since the infection is constantly progressing and it is not easily detectable [9–11]. Several studies have shown that *Chlamydia* migrates to the intestinal tract after infecting the host, develops, and reproduces in the intestinal tract [12, 13]. *Chlamydia* engraftment may alter gut microbial composition and function, affecting host health by inducing gastrointestinal and metabolic diseases [14, 15].

Horses serve as working animals, exhibit animals, or companions during herding activities, and thus are often in close contact with many individuals. Therefore, humans face a higher risk of contracting diseases from horses than other large animals [16]. Although many studies have shown that zoonotic infectious diseases may disturb the gut microbiota of horses [17–19], no study has evaluated gut microbiota changes in horses with *Chlamydia*. Therefore, this study aimed to characterize gut microbiota changes and serum parameters in horses with *Chlamydia* and healthy horses. The results of this study could provide a good foundation for comprehending the pathogenesis of *Chlamydia*-induced intestinal infections in horses, thus safeguarding public health.

## Results

### Serum biochemical indicators of horse of IG and HG

ALB, TP, AST, TG, MDA, SOD, and CAT levels were not significantly different between the IG and HG groups (*P* > 0.05; Table 1). However, GLB, ALT, TC, and CK levels were significantly increased in IG than in HG (Table 1).

**Table 1:** Physiological indicators in IG (n = 10) and HG (n = 6)

Item	IG	HG	SEM	*P* value
ALB (g/L)	30.13	30.12	2.0199	0.998
GLB (g/L)	36.51^a^	28.91^b^	1.4327	< 0.01
TP (g/L)	72.09	65.59	2.3987	0.199
AST (U/L)	219.54	216.68	1.4699	0.364
ALT (U/L)	11.70^a^	8.44^b^	0.4911	< 0.0001
TC (mmol/L)	1.40^a^	1.23^b^	0.0443	< 0.05
TG (mmol/L)	0.44	0.38	0.0689	0.696
CK (U/L)	294.58^a^	179.25^b^	30.7949	< 0.05
MDA (nmol/mL)	2.38	1.81	0.3372	0.426
SOD (U/mL)	66.00	63.64	0.7640	0.826
CAT (U/mL)	45.32	42.30	1.0119	0.155

^a–b^ within rows represents significantly different means (*P* < 0.05). ALB: albumin, GLB: globulin, TP: total protein, AST: aspartate transaminase, ALT: alanine transaminase, TC: total cholesterol, TG: triglyceride, CK: creatine kinase, MDA: malondialdehyde, SOD: superoxide dismutase, CAT: catalase. SEM: standard error of the mean.

## Bacterial community diversity, richness, and OTUs

**Fig. 1:**
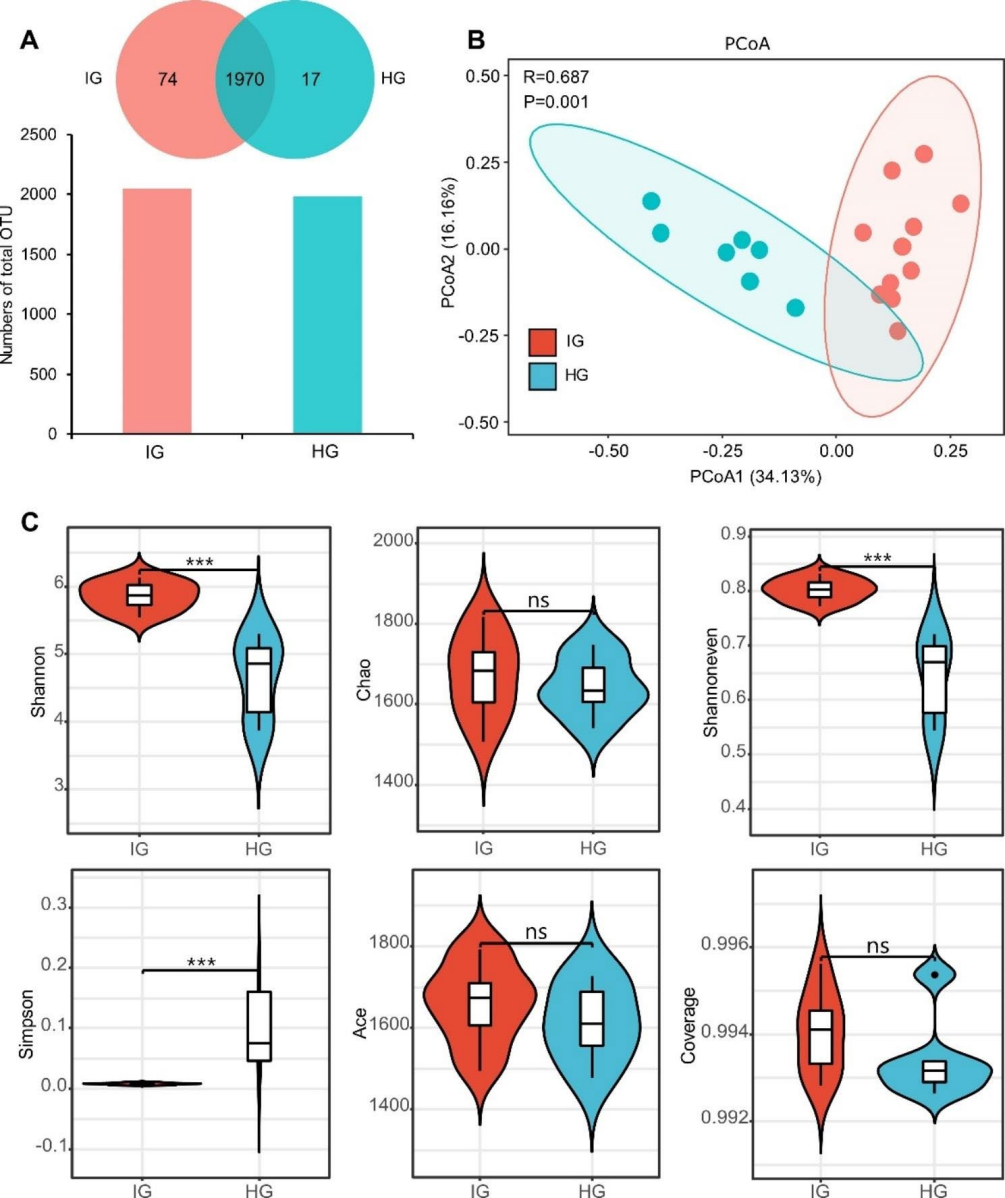
Gut microbial diversity in IG. **A** Venn diagram showing the different and similar OTUs in the IG (n = 10) and HG (n = 6) groups. **B** PcoA showing the OTUs distribution in HG and IG. **C** Shannon index analysis (the gut microbial diversity was significantly increased in IG than in HG) and Simpson index analysis (the gut microbial diversity was significantly decreased in IG than in HG) (* *P* < 0.05, ** *P* < 0.01, *** *P* < 0.001)

A total of 1970 OUT were shared between HG and IG (Fig. 1A). The OUT in the HG and IG were 1987 and 2044, respectively. The samples of HG and IG were distributed discretely and overlapped with each other (*P* < 0.001, *R*^2^ = 0.687), indicating that the samples were reproducible, and HG and IG had different intestinal microorganisms (See Fig. 1B).

The α diversity index analysis showed that the Shannon index and Shannon even index were significantly higher in the IG group than in the HG group (*P* < 0.001). In contrast, the Simpson index was significantly lower in the IG group than in the HG group (*P* < 0.001). Notably, the Chao, Ace, and Coverage indexes (*P* > 0.05) were not significantly different between the HG and IG groups (Fig. 1C).

## Composition of the gut microbiota

**Fig. 2:**
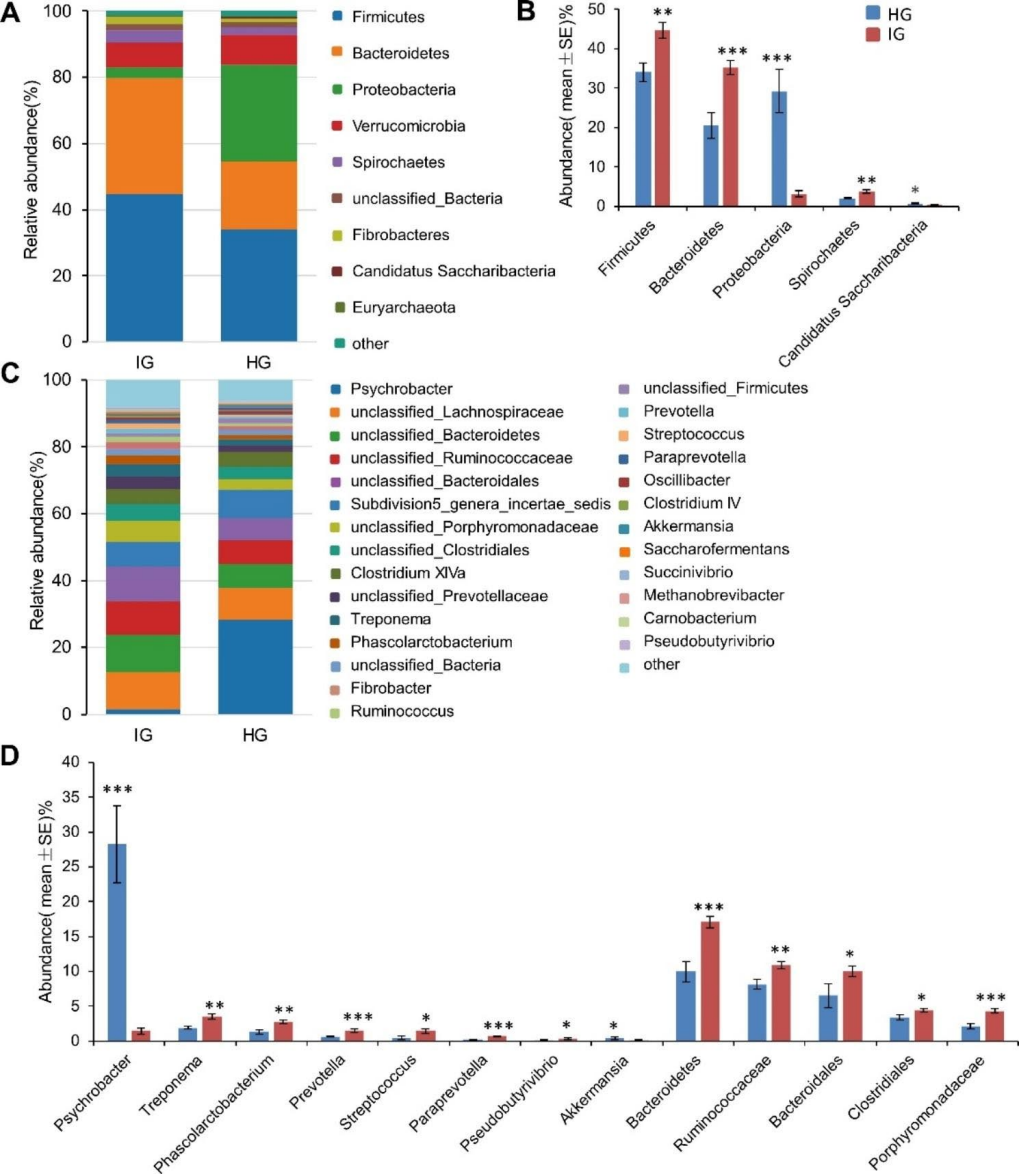
Bacterial compositions in the gut (**A**, phylum; **C**, genus) in HG and IG (HG, healthy groups; IG, Infected groups). The relative abundance of the genus was > 1%, * *P* < 0.05, ** *P* < 0.01, *** *P* < 0.001

The taxa composition and variation of the gut microbiota in HG and IG were analyzed based on the derivation cohort (average relative abundance ≥ 1%). The average composition and relative abundance in gut microbiota at the phylum and genus levels are shown in Fig. 2. *Firmicutes*, *Bacteroidetes*, and *Proteobacteria* were the most predominant phylum in the gut. Compared with HG, the abundance of *Firmicutes* (44.59% vs. 34.01%, *P* < 0.01), *Bacteroidetes* (35.10% vs. 25.22%, *P* < 0.001), and *Spirochaetes* (3.71% vs. 2.03%, *P* < 0.01) was significantly increased in IG, while *Proteobacteria* abundance (7.66% vs. 29.23%, *P* < 0.001) decreased (Fig. 2A and B**)**. Furthermore, the abundance of 5 bacteria, such as *Clostridia*, *Bacteroidia*, *Spirochaetia*, and *Negativicutes* increased in IG, while the abundance of 3 bacteria, including *Gammaproteobacteria* and *Verrucomicrobiae* decreased (*P* < 0.05) (Table S1). The gut microbiota community was also analyzed at the order, family, and genus levels (Fig. 2C, Table S2, and S3). The abundance of 11 bacteria, including *Treponema* (3.47%), *Phascolarctobacterium* (2.75%), *Prevotella* (1.47%), *Streptococcus* (1.42%), *Paraprevotella* (0.66%), *Pseudobutyrivibrio* (0.34%), *Bacteroidetes* (17.06%), *Ruminococcaceae* (10.87%), *Bacteroidales* (9.96%), *Clostridiales* (4.41%), *Porphyromonadaceae* (4.33%) increased in IG at the genus level, while the abundance of two bacteria, including *Psychrobacter* (1.44%) and *Akkermansia* (0.11%) decreased (*P* < 0.05) (Fig. 2C and D).

**Fig. 3:**
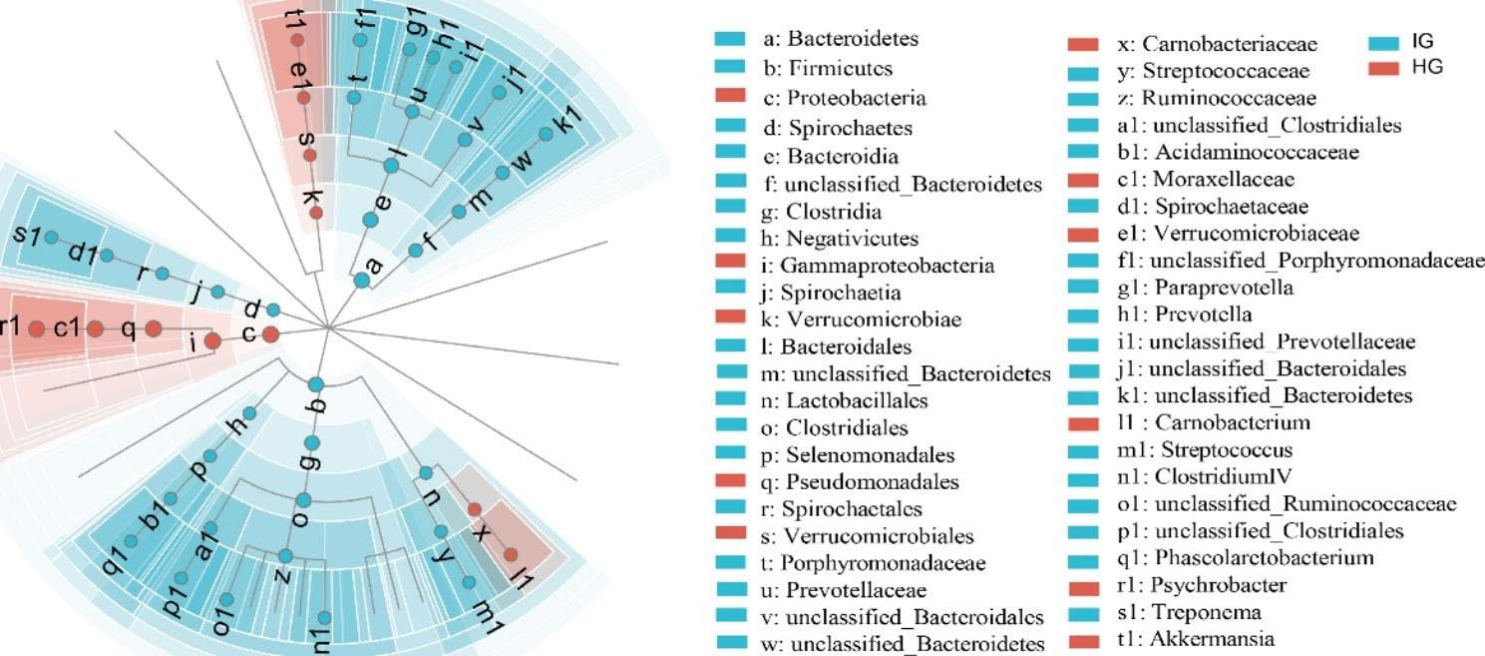
LEFSe cladogram comparing microbial communities between HG and IG horses. The color of the group where taxa are most abundant indicates differences; Red: taxa abundant in HG, Green: taxa abundant in IG.

LEFSe (Linear Discriminant Analysis Effect Size) analysis was conducted to further identify changes in bacterial taxa composition (Fig. 3). LEFSe showed that 11 clades were more abundant in the HG, while 35 clades were more abundant in the IG. The abundance differences are shown in Fig. S2. Notably, the abundance of *Firmicutes* and *Bacteroidetes* was significantly different in IG.

**Fig. 4:**
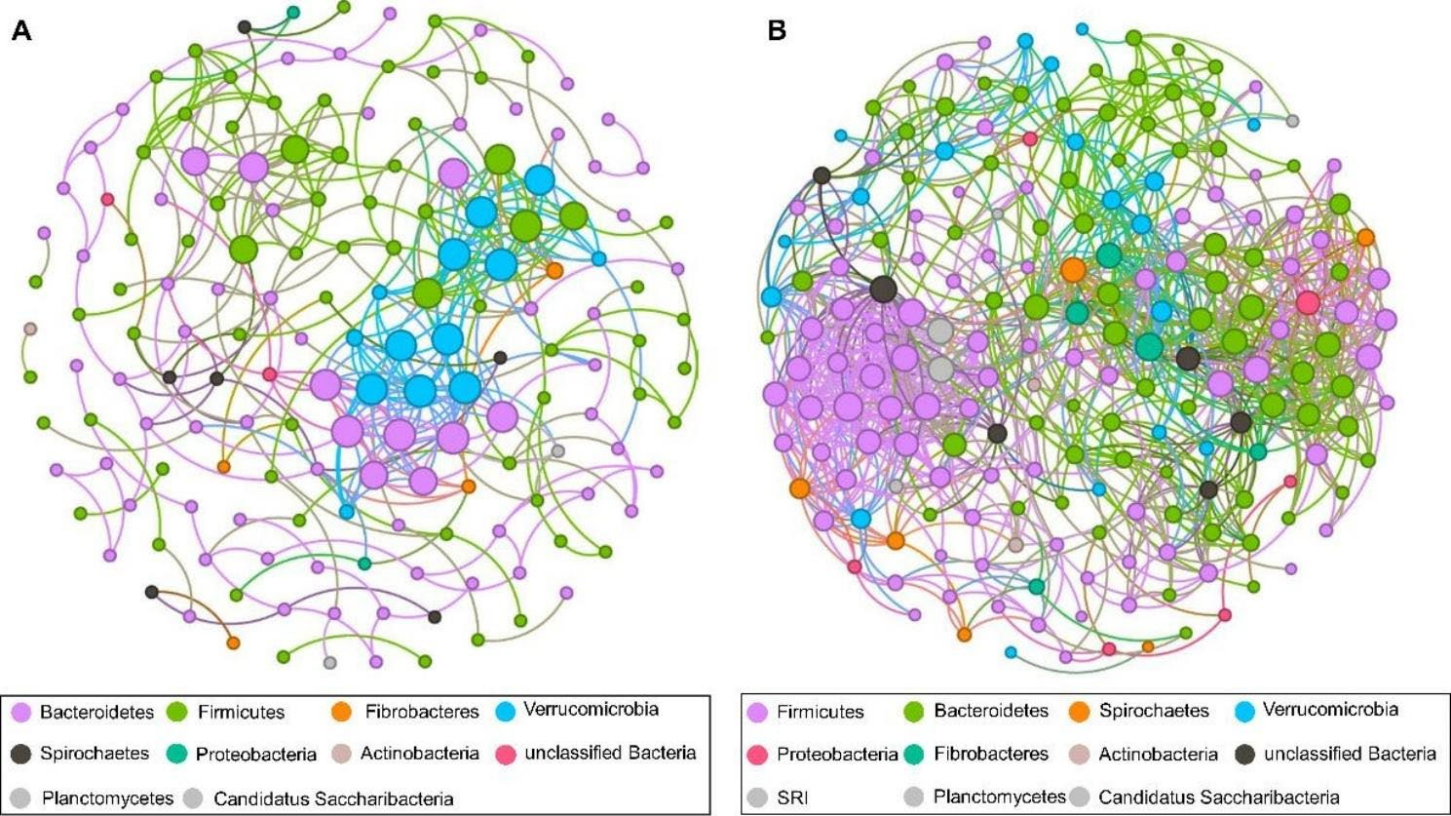
Interaction network of gut microbiota. Correlation network of **(A)** IG and **(B)** HG: 16 S rRNA genes in the gut microbiota of the horse, displaying statistically significant interactions with absolute correlation coefficients > 0.6. Node sizes are scaled according to the overall abundance of each taxon in the microbiota

Network analysis demonstrated that *Chlamydia* infection in horses alters the gut microbial composition and bacterial interactions (Fig. 4). Further results showed that *Bacteroides* were advantageous and core to IG, while *Firmicutes* were advantageous and core to HG. In addition, the network structure of the HG group was more complex, characterized by more nodes and links, compared with the IG group, revealing that *Chlamydia* infection may destabilize gut microbial interactions.

## Prediction of gut microbial gene function

**Fig. 5:**
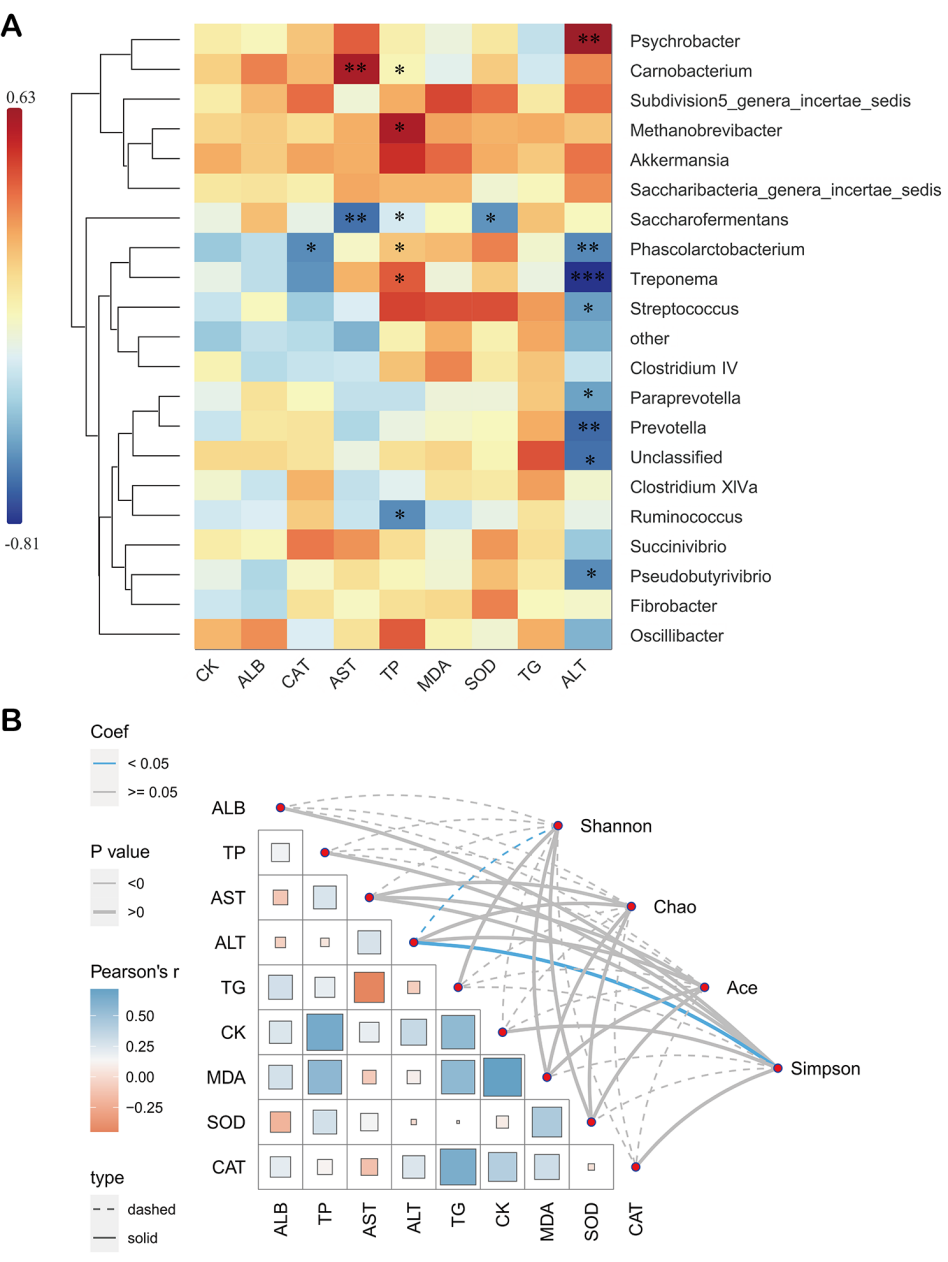
**(A)** Spearman correlation and clustering analysis between serum biochemical indicators and main bacterial at the genus level (* *P* < 0.05, ** *P* < 0.01, *** *P* < 0.001). ALB: albumin, TP: total protein, AST: aspartate transaminase, ALT: alanine transaminase, TG: triglyceride, CK: creatine Kinase, MDA: malondialdehyde, SOD: superoxide dismutase, CAT: catalase. **(B)** Pairwise comparisons of serum indicators: The color gradient represents the Pearson correlation coefficient, and the edge width corresponds to the correlation of Mantel’s statistic. Blue represents a significant correlation, while the solid line represents a positive correlation

Gene function prediction of microbial communities was conducted using PICRUSt software based on the gut microbe 16 S rRNA sequencing data in IG and HG. The differential KEGG pathway analysis showed that 32 pathways were significantly different between the IG and HG groups (*P* < 0.05) (Fig. 5). Specifically, the relative abundances of carbohydrate metabolism, nucleotide metabolism, enzyme families, and biosynthesis of other secondary metabolites significantly increased in IG (*P* < 0.05). Furthermore, the amino acid metabolism pathway (10.78%, *P* < 0.01), lipid metabolism (3.60%, *P* < 0.01), and metabolism of other amino acids (1.67%, *P* < 0.01) were significantly higher in HG than in IG.

## Relationship of serum biochemical indicators with gut bacterial community

A correlation heatmap (correlation threshold > 0.5) of the top 20 species (with high abundances) and serum biochemical indicators (ALB, TP, AST, ALT, TG, CK, MDA, SOD, and CAT) is shown in Fig. 5A. Serum parameters were significantly correlated with the genus (6 positive and 12 negative correlations) (*P* < 0.05). Specifically, *Treponema* was significantly negatively correlated with ALT (*P* < 0.001) and positively correlated with TP (*P* < 0.05). In addition, ALT was significantly negatively correlated with *Prevotella* and *Paraprevotella*, while *Saccharofermentans* was significantly negatively correlated with AST, TP, and SOD (Fig. 5A, P < 0.05). The correlations between α diversity and serum biochemical indicators were also assessed via Pearson’s correlation analysis and mantel test (Fig. 5B). Results showed that ALT was significantly positively and negatively correlated with Simpson and Shannon indices, respectively (Fig. 5B).

**Fig. 6:**
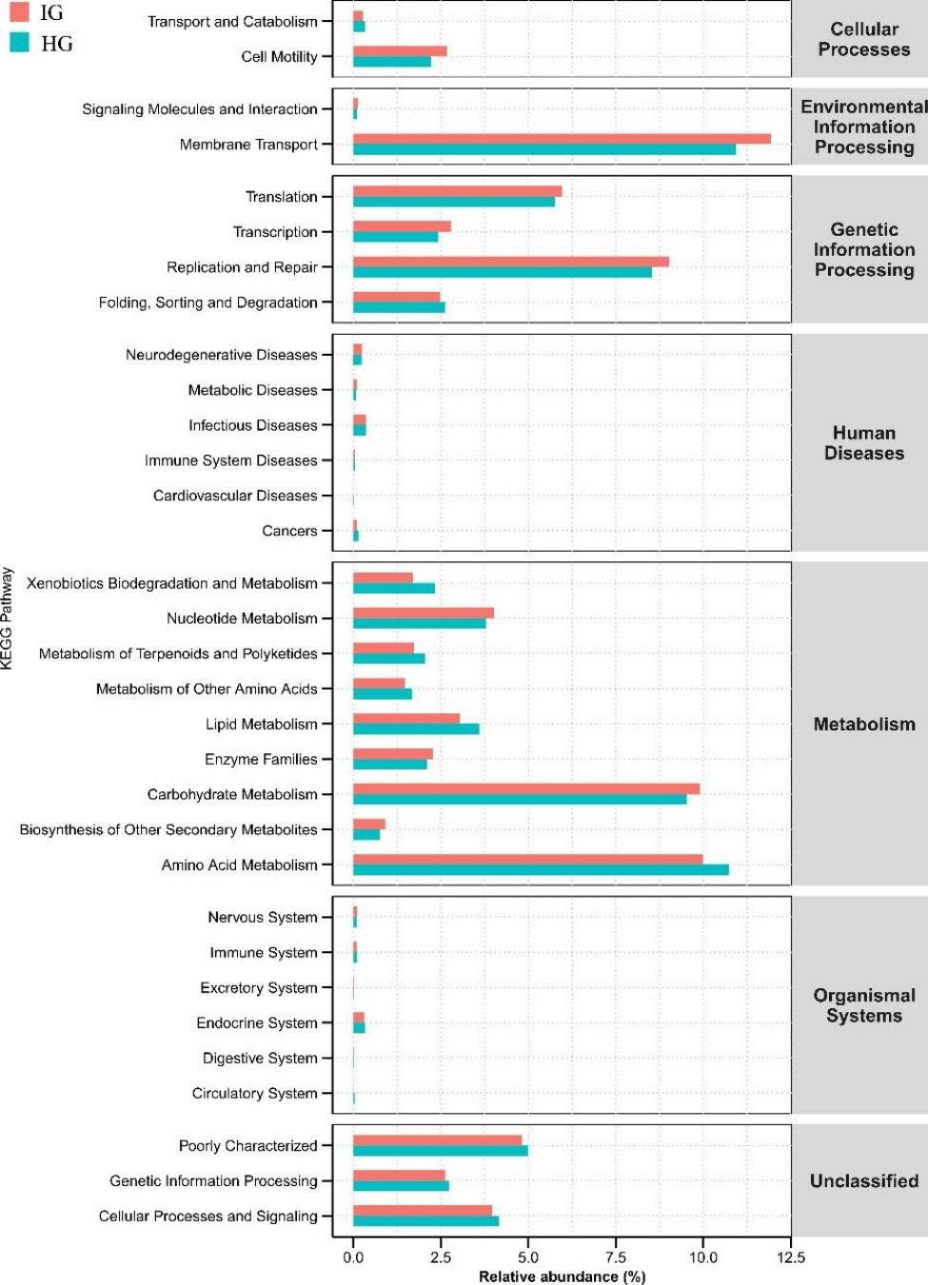
Functional predictions for gut microbiota with significantly different KEGG pathways (*P* < 0.05) for the IG and HG (KEGG pathways at Level 2 and Level 3)

## Discussion

This study elucidates changes in the gut microbiota of horses caused by *Chlamydia* infection. Serum biochemical indicators in the IG and HG group were analyzed and compared. The IG group had higher levels of GLB and ALT (Table 1), similar to a previous human study case [20, 21]. *Chlamydia* is a zoonotic pathogen that invades host cells, which may trigger stress response and increase ALT levels. The *Chlamydia* is characterized by the latent infection of the body and the continuous secretion of cytokines, such as Tumor necrosis factor, interleukin (IL)-1β, and IL-6. Moreover, *Chlamydia* may lead to the continuous damage of multiple organs, such as the liver, kidney, and muscles [22, 23], and increase the permeability of tissue cells, thus increasing ALT levels. Notably, the CK levels were higher in the IG than in HG (Table 1). *Chlamydia* has a unique biphasic developmental cycle [24], with alternating elementary bodies and reticulate bodies. This allows immune escape, leading to persistent infection [25]. CK is widely found in tissues, such as skeletal muscle, cardiac muscle, and brain [26]. *Chlamydia* can hide in monocytes and penetrate the blood-brain barrier after continuous infection, thus spreading to brain tissue [27] and increasing CK levels.

Gut microbes are crucial in the digestion and metabolism of nutrients and the maintenance of host health [28, 29]. *Chlamydia* is a special intracellular pathogen that settles in the gastrointestinal tract, reproductive tract, and other parts after infection, thus changing the ecological niche of the original microbiota [6, 12]. Most studies have focused on the changes in vaginal microbes caused by *Chlamydia* infection, ignoring *Chlamydia* effect on gut microbes [30–32]. Herein, *Firmicutes*, *Bacteroidetes*, and *Proteobacteria* were the most dominant phyla in the horse gut, accounting for about 82.83% and 83.75% of the gut microbiota in HG and IG (Fig. 2A), respectively. [33] also reported similar results in purebred horses. Furthermore, gut microbiota diversity increased in the IG group while bacterial communities were changed (Fig. 1C). Although high microbial diversity may improve the overall metabolic efficiency of the organism, it may also destabilize the system and reduce ecological stability [34]. For example, oral dysbiosis in patients with lupus erythematosus is associated with a more diverse oral microbiota [35]. In addition, increased microbial diversity may be associated with multiple deleterious effects. Similarly, increased microbial diversity is associated with impaired fertility in women in the US [36].

Horses are closely related to humans and there are over 10.5 million horses worldwide (Food and Agriculture Organization of the United Nations 2017) (www.fao.org/faostat/en/#data/QL accessed on June 8, 2023), increasing the risk of cross-species transmission of *Chlamydia* between horses and humans [37]. In this study, *Chlamydia* infection altered the composition of the gut microbiota in horses, indicated by an increased abundance of enteric *Streptococcus*, *Treponema*, *Prevotella*, and *Paraprevotella* (Fig. 2D). *Streptococcus* is the main lactic acid producer. An increased abundance of *Streptococcus* may cause hindgut acidosis in horses, thus exacerbating enteritis [29, 38]. Herein, *Prevotella* and *Paraprevotella* abundances were increased in IG. *Prevotella* and *Paraprevotella* are common bacterial groups in the gut, mainly involved in cellulose degradation and carbohydrate metabolism, producing short-chain fatty acids (propionic acid, butyric acid, acetic acid, etc.). Furthermore, complex interactions occur among gut microbes, such as competition for resources [39] and cross-feeding [40, 41]. Unlike other intestinal opportunistic pathogens, the survival and reproduction of *Chlamydia* in the intestinal environment may require a competitive advantage to accelerate the breakdown of fibers and proteins, thereby increasing the abundance of *Prevotella* and *Paraprevotella* [42].

In the present study, *Moraxellaceae* and *Psychrobacter* dominated the gut microbiota of healthy horses at the order and genus levels (Table S2 and Fig. 2D). Studies have shown that *Moraxellale* and *Psychrobacteriaceae* are common on the mucous membranes of animals and humans [43, 44]. However, their role in resisting *Chlamydia*l infection is unknown. A study found that Moraxella is the dominant nasal microbe in healthy children compared with COVID-19 patients. Reducing *Moraxellaceae* abundance may lead to lipid metabolism and amino acid metabolism disorders [43, 45]. These findings indicate that *Moraxellaceae* may have a special defense mechanism against intracellular infection. Nonetheless, the pathogenic mechanisms of COVID-19 and *Chlamydia* are different. Therefore, further studies should assess Moraxella in cell models infected with *Chlamydia*. In this study, the abundance of *Akkermanisa* was significantly reduced in the IG group. *Akkermanisa* is a promising next-generation probiotic [46], which can effectively improve intestinal inflammation, reduce the secretion of inflammatory cytokines, and protect intestinal health [47]. Therefore, *Akkermanisa* may be used as a new biological agent for the treatment of enteritis caused by *Chlamydia* infection.

Microorganisms play a key role in immunity, nutrient absorption, and enzyme metabolism [48]. Results showed that carbohydrate metabolism in the gut microbiota was enhanced in the IG group. Carbohydrate provides energy that is easily metabolized and utilized by the body [49]. *Chlamydia* requires a lot of energy to support its colonization and development after infection and thus may stimulate the utilization of carbohydrates in the body [50, 51]. Herein, lipid metabolism and amino acid metabolism were reduced in IG (Fig. 6). Although lipid metabolism can also be used as an energy source for the body, *Chlamydia* infection causes cellular immune responses, then produces inflammatory responses and releases cytokines [52, 53], thereby inhibiting lipid metabolism and amino acid metabolism. Furthermore, the inflammatory response can inhibit the synthesis and oxidation of fatty acids while increasing the uptake and utilization of glucose [54], thus enhancing carbohydrate metabolism [55]. However, these results were only based on the predicted metagenomics and may not represent the actual function of gut bacteria. Therefore, future studies should explore the role of these genes in the gut of horses with *Chlamydia* based on metabolomics.

Although the changes in gut microbes in horses infected with *Chlamydia* were comprehensively revealed, *Chlamydial* infection is usually accompanied by an invisible infection. Therefore, the influence of several stages of *Chlamydial* occurrence, development, and identification on the intestinal tract should be comprehensively analyzed. In addition, the number of horses should be increased in future studies to avoid the influence of environmental and dietary factors. Future studies should also incorporate continuous monitoring of large sample sizes, combining metabolomics and microbiome multiple combinations to reveal the intestinal pathogenesis caused by *Chlamydia* infection.

## Conclusion

In summary, the changes in serum biochemical parameters and gut microbiota in feedlot horses with *Chlamydia* infection were evaluated. *Chlamydia* is an intracellular pathogen that settles in the intestinal tract after infection, increasing the diversity of intestinal microorganisms. Herein, the gut microbiota was correlated with serum biochemical markers of *Chlamydial* infection. Moreover, the gut microbiota changed with increasing disease activity. These results suggest that gut microbiota dysbiosis may promote the development of *Chlamydial* infection. Interestingly, *Akkermanisa* was more abundant in the gut of HG than in the IG group, revealing that this family may protect against *Chlamydial* infection.

## Materials and methods

### Animal care

The study was conducted following the guidelines of the Administration for Experimental Animal Affairs (Ministry of Science and Technology, China; revised in June 2024). Moreover, the samples collection and the study were approved by the Animal Use and Care Ethics Committee of Lanzhou University (Gansu, China, No. 2010-1 and 2010-2).

### Experimental site

The study was conducted in March 2023 at a horse farm in Shandan County (Zhangye City, Gansu Province). The area has an altitude of ≈ 2500 m, an annual average temperature of 0 °C, and a precipitation of 360 mm. The grassland type in the area is known as an alpine meadow based on the comprehensive, sequential classification method. The vegetation types in the area mainly include *Gramineae*, *Cyperaceae*, *Fabaceae, Compositae*, and *Rosaceae*.

### Animal husbandry and samples

The horses were mixed bred with horses grazing in summer and housed while feeding in winter. The horses had the same diet and were free to drink water and lick brick salt. Moreover, the horses had free access to light and the external environment (temperature and activity space).

However, the horses were reported to experience abortion. A surveillance study was conducted to examine the epidemiology of abortive pathogens. Briefly, fecal samples were collected from the interior of the anus in the early morning (at 09:00 h) without stimulation for three consecutive days and immediately placed in 50 mL sterile propylene tubes with a filter cap. The samples were immediately stored at -20 °C, and transported on ice to the State Key Laboratory of Livestock Diseases, Lanzhou Veterinary Research Institute, Chinese Academy of Agricultural Sciences.

Blood samples were also collected through the jugular vein before morning feeding and centrifuged at 3000 rpm at 4 °C for 15 min to obtain serum. The serum was separated and placed in a 2 mL centrifuge tube at -20 °C for subsequent analysis.

### *Chlamydia* testing and serum biochemical assays

A fluorescent quantitative PCR (qPCR) was used to detect the pathogens causing miscarriage. The primers and probes used in this analysis are listed in Table S1. The reaction system (20 µL) contained 6 µL of dd H_2_O, 0.8 µL of upstream primer and downstream primer each, 10 µL of 2×Quantinova Green PCR Master Mix, and 2 µL of the sample. Positive and negative controls were also set up. The program detection was conducted as follows: 45 cycles of pre-denaturation at 95 °C for 3 min, denaturation at 95 °C for 30 s, and annealing at 55 °C for 30 s. The CT value < 35 was considered positive during the preparation of positive and negative controls (Fig. S1).

Epidemiological and clinical investigations detected *Chlamydia* infection (Fig. S1), with an incidence rate of 57.87% (unpublished data). Sixteen healthy Shandan mares (48 months old) were randomly selected from a herd housed in the same barn for further study to avoid confounding factors, such as environmental factors and other diseases. The horses were divided into two groups: 6 into the uninfected (HG) horses and 10 into the *Chlamydia*-infected horses (IG).

Albumin (ALB), globulin (GLB), total protein (TP), aspartate transaminase (AST), alanine transaminase (ALT), total cholesterol (TC), triglyceride (TG), creatine kinase (CK), malondialdehyde (MDA), superoxide dismutase (SOD), catalase (CAT) using the respective kit (Jiangsu Jingmei Biological Technology Co., Ltd, China).

### Deoxyribonucleic acid (DNA) extraction, amplification, and sequencing

Library construction and sequencing of specific amplification products were completed by Sangon Bioengineering Co., Ltd. Target sequence amplification was conducted via a two-step PCR method (compatible with Illumina sequencing library preparation). The volume of the first round of the PCR system (25 µL) contained 2 µL DNA template, 1 µL upstream primer, 1 µL downstream primer, and 15 µL 2×PCR Ready Mix. The PCR execution program was as follows: pre-denaturation at 98 °C for 3 min, followed by 8 cycles of denaturation at 98 °C for 30 s, annealing at 60 °C for 30 s, extension at 72 °C for 30 s, and extension at 72 °C for 5 min. The PCR product was recovered using AMPure XP magnetic beads after the size of the product was confirmed. The second round of PCR reaction then obtained a sequenced tagged molecular library. Briefly, the reaction system (30 µL) contained 2 µL DNA template, 1 µL P7 primer with molecular tag, 1 µL P5 primer with molecular tag, and 15 µL 2×PCR Ready Mix. The PCR reaction program was conducted as follows: pre-denaturation at 98 °C for 5 min, followed by five cycles of denaturation at 94 °C for 30 s, annealing at 55 °C for 30 s, extension at 72 °C for 30 s, and extension at 72 °C for 5 min. Notably, the PCR products were recovered using AMPure XP magnetic beads, while NovaSeq6000/ MiSeq sequencer (Illumina, San Diego, CA) was used for paired-end sequencing (150 bp/300 bp).

The high-throughput sequencing data first underwent quality control. Second, the linker sequence was excised using Cutadapt (v 1.2.1) software, while the positive and negative sequences of the same sequence were spliced using Pear (v 0.9.6) software. The sliding window method was used to remove low-quality base sequences via Pinseq-lite (v 0.19.5) software (the minimum Q value; 20, window value; 5, and length threshold; 50). Chimeric sequences were further removed using the search (v 11.06.66) software in de novo mode. Finally, the target sequence was matched according to the upstream and downstream primers through the Python program. Multiple sequence alignment was performed.

### Statistical analysis

The Serum biochemical indicators (ALB, GLB, TP, AST, ALT, TC, TG, CK, MDA, SOD, and CAT) and alpha diversity indexes (Ace Index, Chao1 Index, Shannon Index, Simpson Index, Shannon even Index, Sobs and Coverage Index) were analyzed using a completely randomized design based on one-way analysis of variance (ANOVA) (version 24.0, software SPSS Inc., Chicago, IL, USA). A significant tendency was detected at 0.01 ≤ *P* ≤ 0.05. Key taxa of the microbial community were identified using a microbial network based on a composite score of high mean, high proximity centrality, and low betweenness centrality. The correlation between the bacterial genera and Serum biochemical indicators was evaluated using a Spearman correlation test.

### Electronic supplementary material

Below is the link to the electronic supplementary material.

Supplementary Material 1

## Data Availability

The datasets presented in this study can be found in online repositories. The name of the repository and accession number can be found below: NCBI; PRJNA996933.
