# Supplementary material for: Gut microbiota changes in horses with Chlamydia
Source: BMC Microbiol. 2023 Sep 2;23:246. doi: 10.1186/s12866-023-02986-8 (PMC10474637; doi:10.1186/s12866-023-02986-8)
Supplement: Supplementary file 1 — Supplementary Material 1 [file 12866_2023_2986_MOESM1_ESM.docx]

**Table S1** *Chlamydia* species Quantitative Real-time PCR primers and probes

| Species | Primer and probe | Sequences（5’~3’） |
| --- | --- | --- |
| *Chlamydiaceae* | Forward | CTGAAACCAGTAGCTTATAAGCGGT |
|  | Reverse | ACCTCGCCGTTTAACTTAACTCC |
|  | Probe | FAM-CTCATCATGCAAAAGGCACGCCG-BHQ1 |


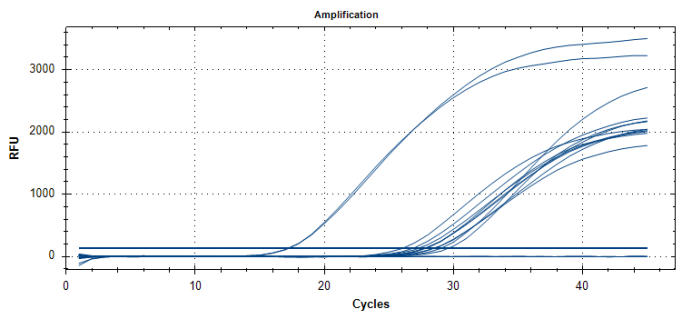


**Fig. S1.** *Chlamydia* infection in horses detected by qPCR

**Table S2** Bacterial comparisons of the gut in IG and HG. The relative abundance of class was obtained to be > 1%

| Item | HG | IG | *P*-value |
| --- | --- | --- | --- |
| Clostridia | 29.351 | 37.8364 | **0.02** |
| Bacteroidia | 12.8829 | 23.5225 | **0.001** |
| Gammaproteobacteria | 28.5914 | 2.2756 | **0** |
| unclassified_Bacteroidetes | 7.2082 | 11.052 | **0.006** |
| Subdivision5 | 8.409 | 7.4408 | 0.736 |
| Spirochaetia | 2.0263 | 3.708 | **0.007** |
| Negativicutes | 1.6367 | 3.0473 | **0.009** |
| Bacilli | 1.1806 | 2.239 | 0.061 |
| unclassified_Bacteria | 1.6384 | 1.8039 | 0.57 |
| Fibrobacteria | 0.9813 | 2.0655 | 0.15 |
| unclassified_Firmicutes | 1.4631 | 0.9219 | 0.085 |
| norank_Candidatus Saccharibacteria | 0.6689 | 0.2504 | **0.021** |
| Verrucomicrobiae | 0.7486 | 0.1967 | **0.04** |
| Methanobacteria | 0.3127 | 0.2457 | 0.716 |
| other | 2.9003 | 3.3935 | 0.142 |

**Table S3** Gut microbial composition at order level in IG and HG

| Item | HG | IG | *P*-value |
| --- | --- | --- | --- |
| Clostridiales | 29.1115 | 37.508 | **0.02** |
| Bacteroidales | 12.8829 | 23.5225 | **0.001** |
| Pseudomonadales | 28.2058 | 1.5012 | **0** |
| unclassified_Bacteroidetes | 7.2082 | 11.052 | **0.006** |
| norank_Subdivision5 | 8.409 | 7.4408 | 0.736 |
| Spirochaetales | 2.0263 | 3.708 | **0.007** |
| Selenomonadales | 1.6367 | 3.0473 | **0.009** |
| unclassified_Bacteria | 1.624 | 1.7887 | 0.567 |
| Fibrobacterales | 0.9813 | 2.0655 | 0.15 |
| Lactobacillales | 0.8833 | 2.1127 | **0.034** |

**Table S4** Gut microbial composition at family level in IG and HG

| Item | HG | IG | *P*-value |
| --- | --- | --- | --- |
| Lachnospiraceae | 14.9034 | 17.7344 | 0.221 |
| Ruminococcaceae | 9.9046 | 14.1309 | **0** |
| Moraxellaceae | 28.122 | 1.4307 | **0** |
| unclassified_Bacteroidetes | 7.2082 | 11.052 | **0.006** |
| unclassified_Bacteroidales | 6.6315 | 10.2869 | **0.031** |
| norank_Subdivision5 | 8.409 | 7.4408 | 0.736 |
| Porphyromonadaceae | 3.3234 | 6.7411 | **0.001** |
| Prevotellaceae | 2.9035 | 6.4578 | **0.013** |
| unclassified_Clostridiales | 3.8027 | 5.0565 | **0.04** |
| Spirochaetaceae | 2.0263 | 3.7073 | **0.007** |
| Acidaminococcaceae | 1.295 | 2.7411 | **0.005** |
| unclassified_Bacteria | 1.6251 | 1.7912 | 0.564 |
| Fibrobacteraceae | 0.9813 | 2.0655 | 0.15 |
| unclassified_Firmicutes | 1.4631 | 0.9219 | 0.085 |
| Streptococcaceae | 0.4216 | 1.4151 | 0.055 |
| norank_Candidatus Saccharibacteria | 0.6689 | 0.2504 | **0.021** |
| Verrucomicrobiaceae | 0.7486 | 0.1967 | **0.04** |
| Succinivibrionaceae | 0.0891 | 0.5186 | 0.339 |
| Methanobacteriaceae | 0.3127 | 0.2457 | 0.716 |
| Carnobacteriaceae | 0.3372 | 0.2516 | 0.727 |
| other | 4.8221 | 5.5626 | 0.088 |


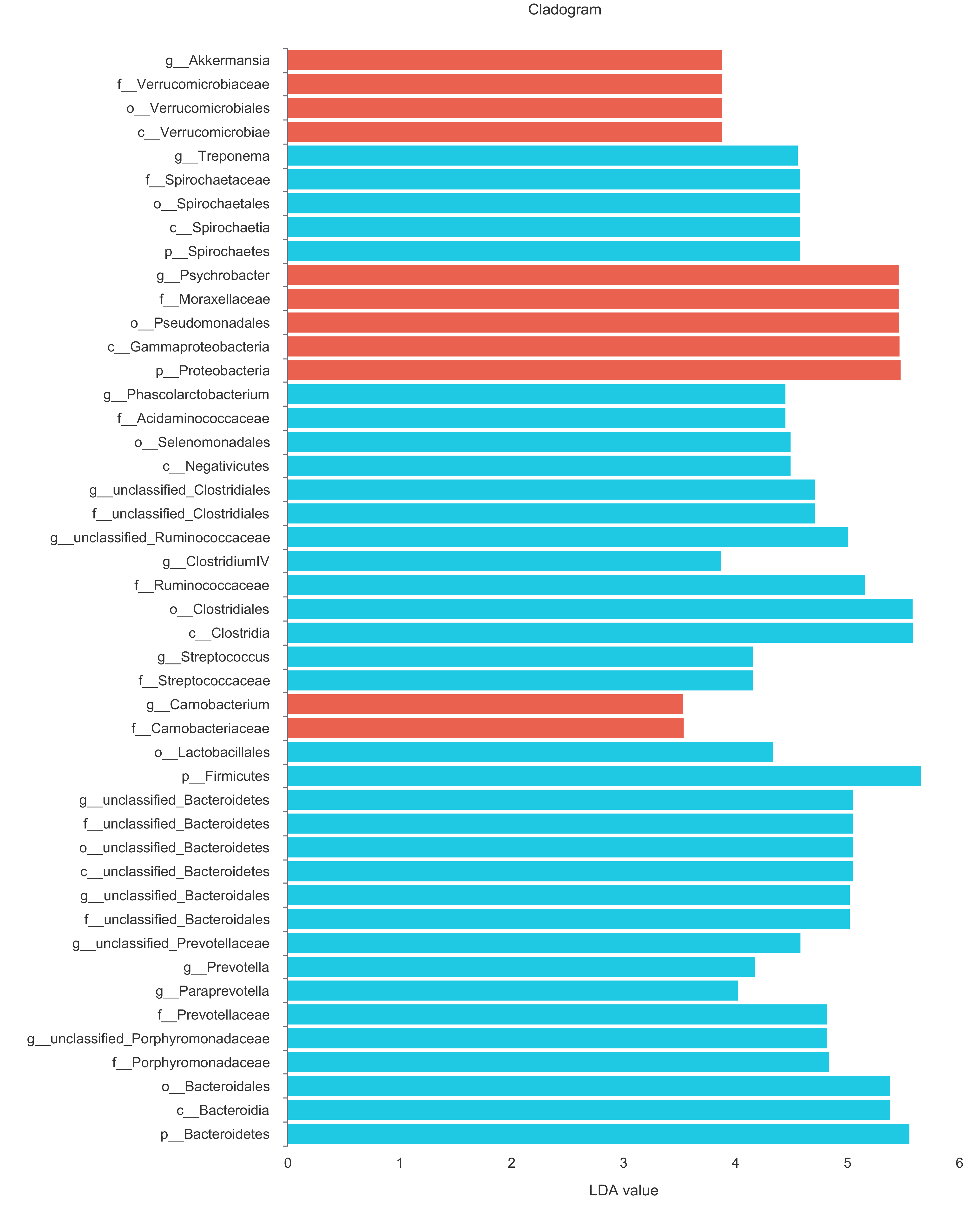


Fig. S2 Histogram of LDA score calculated for each taxon ranging from phylum to genus.
